# TYPOLOGY OF FAMILY RELATIONSHIP AND ELDER MISTREATMENT IN A U.S. CHINESE POPULATION

**DOI:** 10.1093/geroni/igz038.2794

**Published:** 2019-11-08

**Authors:** Mengting Li, Man Guo, Meredith Stensland, Merril Silverstein, XinQi Dong

**Affiliations:** 1 Rutgers, The State University of New Jersey, New Brunswick, New Jersey, United States; 2 University of Iowa, Iowa City, Iowa, United States; 3 University of Texas at Austin, Austin, Texas, United States; 4 Syracuse University, Syracuse, New York, United States

## Abstract

Early research on family relationship and Elder Mistreatment (EM) often focused on one or two indicators of relations. A typology approach that capture the complexity and variation of relations is a useful tool to understand the association between multifaceted family relationship and EM. EM was measured by a modified Vulnerability to Abuse Screening Scale. Latent Class Analysis was used to construct family typologies, evaluating structural, associational, functional, affectual, and normative aspects of family relationship. Logistic regression was used. Unobligated ambivalent (OR, 1.90; 95%CI, 1.54-2.34) and detached (OR, 1.78; 95%CI, 1.32-2.42) typologies were associated with greater risk of EM, while tight-knit (OR, 0.34; 95%CI, 0.27-0.44) typology was associated with lower risk of EM. Unobligated ambivalent typology, featured by high intergenerational closeness and conflict, was prevalent among US Chinese immigrants, and associated with greater likelihood of EM. Culturally customized social services were suggested to reduce intergenerational ambivalence and prevent EM for immigrants.

